# Currarino Triad: Importance of Preoperative Magnetic Resonance Imaging

**DOI:** 10.1055/s-0039-3399533

**Published:** 2019-11-22

**Authors:** Amr AbdelHamid AbouZeid, Shaimaa Abdelsattar Mohammad, Mohammad Seada, Khaled Khiamy, Radwa Gamal

**Affiliations:** 1 Department of Pediatric Surgery, Faculty of Medicine, Ain Shams University, Cairo, Egypt; 2 Department of Radiodiagnosis, Ain Shams University, Cairo, Egypt; 3 Department of Pediatric Surgery, Benha Specialized Children Hospital, Benha, Egypt; 4 Genetics Unit, Department of Pediatrics, Ain Shams University, Cairo, Egypt

**Keywords:** Currarino triad, presacral cyst, anorectal malformation, MRI

## Abstract

Currarino triad is a rare syndrome that may be occasionally encountered during managing cases of anorectal anomalies. The triad consists of anorectal anomaly, sacral bony defect, and a presacral mass. It may be familial or sporadic, with a reported female predominance. Identification of the characteristic notched sacrum (sacral scimitar) in plain X-ray (anteroposterior view) is considered the key for the diagnosis; however, not infrequently, this radiological sign is overlooked, especially with a small sacral defect.

Excision of the presacral cyst is usually performed concomitantly during anorectoplasty. The prone position is the standard approach for posterior sagittal anorectoplasty (PSARP) in males; however, in females, the supine position can be used as an alternative (anterior sagittal anorectoplasty). In this case report, excision of the presacral cyst took place in two steps: the first excision during the PSARP procedure in the prone position, and a second operation in the supine lithotomy position to remove a residual component of the lesion that was missed during the primary operation. It was clear that the supine lithotomy position provided better access to explore the presacral space than the prone position, especially with a deeply located cyst as in our case. The role of magnetic resonance imaging (MRI) in the identification of the exact nature and extent of the lesion before surgery is crucial and should be performed in all cases.

## Introduction

Currarino triad is a rare congenital disorder characterized by the presence of three components: anorectal anomaly, sacral vertebral defect, and a presacral mass (lipomyelomeningocele and/or developmental cyst). 1 2 Cases may present with the complete form of the triad or may lack one of the three components. 3 Unlike other forms of sacral agenesis, Currarino triad is characterized by partial dysplasia sparing the first sacral vertebra. 1 4 The characteristic sacral defect (sacral scimitar) is considered the key for making the diagnosis. 5

Early reports in the literature were described by Kennedy 6 and Ashcraft and Holder. 7 However, the triad was named after an American radiologist “Guido Currarino,” who was the first to describe the triad as a unique syndrome with characteristic genetic inheritance and provided embryological explanation for its occurrence. 1 Increased awareness of this rare condition can help in achieving early detection, allowing for a more effective way of management.

## Case Presentation

**Fig. 1:**
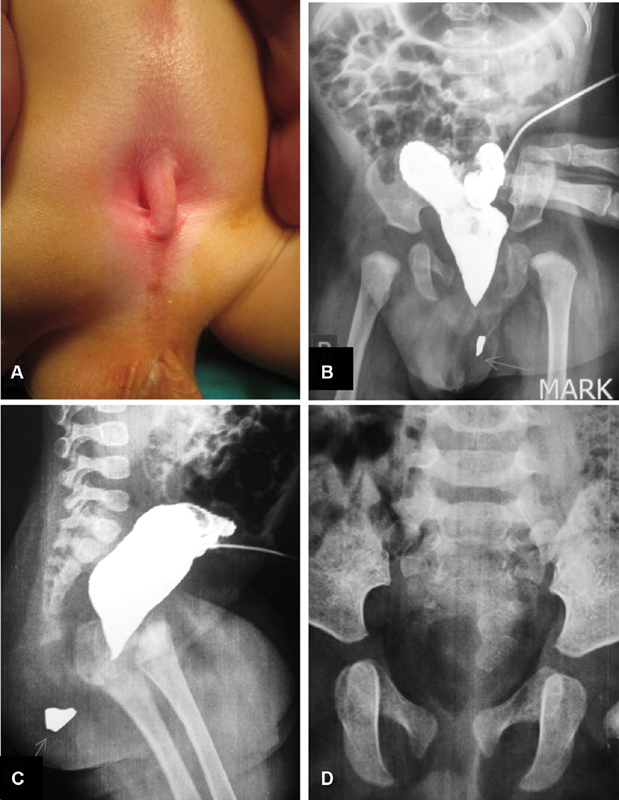
( **A** ) A 3-month-old boy with anorectal anomaly underwent colostomy at birth. ( **B,C** ) The contrast study “distal colostogram” performed in anteroposterior and lateral views showing blind rectal termination with no evidence of fistulous communication with the urinary tract (imperforate anus without fistula). Note: a radio-opaque mark has been placed indicating the site of anal dimple on the perineum ( *arrow* ). ( **D** ) Anteroposterior view of the plain X-ray showing the characteristic sacral defect (sacral scimitar). Note: the sacral notch cannot be detected in the lateral view of sacrum ( **C** ) and has been masked by the contrast in the anteroposterior view ( **B** ).

A male patient presented at birth with anorectal malformation ( Fig. 1A ); there were no signs of meconium staining in the urine or the perineum. A divided pelvic colostomy with distal mucous fistula was performed. Echocardiogram and abdominal ultrasound were negative for possible associated cardiac and renal anomalies.

At the age of 3 months, a high-pressure distal colostogram was performed as a preparatory step for a PSARP (posterior sagittal anorectoplasty) procedure. 8 The distal colostogram demonstrated a blind rectal termination with no evidence of fistulous communication with the urinary tract (imperforate anus without fistula; Fig. 1B, C ).

At the time of operation, the mother revealed that her elder daughter (now 6 years old) underwent a similar operation. Revising the medical records of her daughter revealed a case of Currarino triad: anal stenosis (funnel anus), vertebral defect, and presacral cyst. Because of the reported autosomal dominant inheritance in cases of Currarino triad, 1 9 we suspected that this boy might have a similar pathology. Reexamination of his X-ray studies revealed a missed sacral notch in the plain X-ray ( Fig. 1D ) in addition to widening of the retrorectal space in lateral contrast film ( Fig. 1C ). The diagnosis of familial Currarino triad was quite evident in this boy (like his sister). A decision had to be made whether to postpone the operation and wait for a preoperative pelvic magnetic resonance imaging (MRI) study (for better delineation of the anatomy) or to proceed with the PSARP procedure and explore the presacral space during operation. Influenced by the busy operative schedule and the author's previous surgical experience with similar cases, 5 we decided to operate.

With the patient in the prone position, a generous posterior sagittal incision was made for mobilization of the anorectum and exploration of the presacral space. We started as usual by dissection of the fascia around the posterior and the lateral aspects of the rectal pouch. A small midline incision was made in the posterior wall of the rectum to inspect for any missed communication with the urinary tract and to guide the dissection during separation of the anterior rectal wall from the intimately adherent urinary tract. After mobilization of the anorectum, a retrorectal cyst (∼2 cm in diameter) was found and excised. Then, the steps of anorectoplasty were completed as usual by reconstruction of the levator ani and striated muscle complex around the neorectum.

Postoperative recovery was uneventful. Histopathological examination of excised specimen revealed fibromuscular tissue showing multiple clefts lined by stratified squamous epithelium and entrapped transitional epithelial islands. These histopathological findings go with the diagnosis of a developmental cyst associating with Currarino triad. 10 11 12

**Fig. 2:**
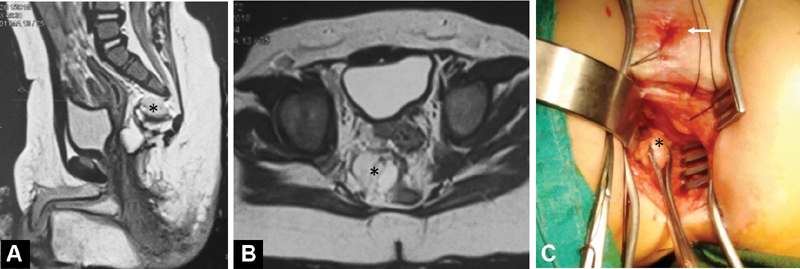
Follow-up magnetic resonance imaging (MRI) study performed after posterior sagittal anorectoplasty showing a residual presacral mass (*) in midsagittal T2-weighted image ( **A** ) and axial T2-weighted image ( **B** ). ( **C** ) Reoperation in the supine lithotomy position to remove the residual presacral mass (*); note: the incision is posterior to the neoanus ( *white arrow* ).

Later, a postoperative pelvic MRI was ordered that revealed residual presacral cystic lesion ( Fig. 2A, B ), with the absence of spinal cord anomalies. The patient was scheduled for reoperation to excise the residual presacral mass while still having a diverting colostomy. This time, the patient was positioned in the supine (lithotomy) position, which provided better exposure to the presacral space. Initially, the lesion was obscured by its deep location and surrounding fat; however, deeper dissection directed toward the bony defect (sacral notch) enabled successful identification and excision of the lesion ( Fig. 2C ). Although the preoperative MRI did not identify any communication between the lesion and the thecal sac, yet careful closure of the bed of the excised lesion was followed. Again, we had uneventful postoperative recovery and histopathological analysis of the excised specimen returned similar to the previous one. Six weeks later, the patient underwent colostomy closure.

The patient was seen at follow-up 3 months after the closure of colostomy. He defecates spontaneously once every 1 to 2 days. He is still only 19 months old and therefore below the age of voluntary bowel control. However, the mother was informed about the possibility of constipation, problems with fecal continence, and the need for long-term follow-up. The mother has had a similar experience with her elder daughter who is now 6 years old and has voluntary bowel and urinary control. The parents (being the first-degree relatives of our case) were advised to perform screening by plain X-ray sacrum.

## Discussion

Currarino triad is a rare syndrome that may be occasionally encountered during managing cases of anorectal anomalies. The triad consists of anorectal anomaly, sacral bony defect, and a presacral mass. 1 It has a reported female predominance and genetic inheritance. Identification of the characteristic notched sacrum (sacral scimitar) in plain X-ray (anteroposterior view) is considered the key for the diagnosis; however, not infrequently, this radiological sign is overlooked, especially with a small sacral defect and a constipated child. 5 Increased awareness about this condition among neonatologists and pediatric surgeons can help in early picking of affected cases and better planning of surgical treatment.

Currarino triad is caused by heterozygous mutation in the *MNX1* (previously *HLXB9* ) gene on chromosome 7q36. The disorder is an autosomal dominant genetic trait that has variable expressivity. 9 Mutations in the coding sequence of the *MNX1* gene have been identified in nearly all cases of familial Currarino's syndrome and in approximately 30% of patients with sporadic Currarino's syndrome. 13 Lynch et al. reported that 4% of patients with a mutation in the gene were asymptomatic and had normal sacral X-ray imaging. 14 Cytogenetic analysis of a previously reported family with Currarino triad showed that the mother of the two affected children carried a balanced translocation between chromosome 7q36 and 12q24. Both children were monosomic for 7q36, as they had inherited the deleted chromosome 7 from their mother. 15

All affected families should be offered genetic counseling, as awareness of the hereditary nature of the disorder allows identification of asymptomatic heterozygotes and patients at risk, which leads to better planning of pregnancies and appropriate management of affected patients. All first-degree relatives of patients with Currarino triad should be screened by pelvic X *-* ray due to a highly variable phenotype within most families. 9 Relatives with an abnormal X *-* ray should be referred to a surgeon for further investigations, including pelvic MRI for an occult presacral mass due to significant inter- and intrafamilial variability in expression. 9 14

The presacral mass in Currarino triad may be a lipoma, lipomyelomeningocele, or some sort of developmental cyst. 1 2 5 The latter has been frequently described in the literature as a mature cystic teratoma, which has been considered by Weinberg to be a misnomer in cases of Currarino triad. 12 There are several observations concerning the presacral cysts associating with Currarino triad that would support the diagnosis of a developmental cyst rather than a true neoplasm (teratoma). 11 It is usually recommended to excise presacral developmental cysts as these are liable for infection and abscess formation. 5 On the other hand, presacral lipomas and meningoceles can be managed conservatively unless symptomatic. 5 Large lesions may cause obstructive symptoms (constipation) and may distort the surgical field during the repair of anorectal anomaly in addition to other related obstetric considerations. 5

Excision of the presacral cyst is usually performed concomitantly during anorectoplasty. The prone position is the standard approach for PSARP in males; 8 however, in females, the supine position can be used as an alternative (anterior sagittal anorectoplasty). 16 In this case report, excision of the presacral cyst took place in two steps: the first excision during the PSARP procedure in the prone position, and a second operation in the supine lithotomy position to remove a residual component of the lesion that was missed during the primary operation. It was clear that the supine lithotomy position provided better access to explore the presacral space than the prone position, especially with a deeply located cyst as in our case. Other lessons learned from managing this case were the importance of proper history taking in surgical practice, possible association of Currarino triad with different types of Anorectal anomalies (not necessarily the “funnel” anus or rectoperineal anomalies), and, lastly, the necessity of performing preoperative MRI to complete the diagnosis in cases of anorectal anomalies associated with sacral defects, which might have spared our case a second operation.

There was some delay initially in completing the diagnosis for this case; however, the management was completed with a satisfactory outcome. Lastly, the prognosis for continence may be generally good for this case owing to the absence of associated spinal cord anomalies. 5

## Notes

New Insights and the Importance for the Pediatric Surgeon Currarino triad is a rare syndrome that may be occasionally encountered during managing cases of anorectal anomalies. Increased awareness of this rare condition can help in achieving early detection, allowing for a more effective way of management.
